# Metagenomic CRISPR Array Analysis Tool: a novel graph-based approach to finding CRISPR arrays in metagenomic datasets

**DOI:** 10.1093/femsml/uqaf016

**Published:** 2025-07-17

**Authors:** Fikrat Talibli, Björn Voß

**Affiliations:** University of Stuttgart, Institute of Biomedical Genetics, Department of RNA Biology and Bioinformatics, Allmandring 31, 70569 Stuttgart, Germany; University of Stuttgart, Institute of Biomedical Genetics, Department of RNA Biology and Bioinformatics, Allmandring 31, 70569 Stuttgart, Germany

**Keywords:** CRISPR-Cas, CRISPR array, metagenomic, bioinformatics, de Bruijn graph, cycles

## Abstract

Clustered Regularly Interspersed Short Palindromic Repeats and CRISPR-associated genes (CRISPR-Cas) is a bacterial immune system also famous for its use in genome editing. The diversity of known systems could be significantly increased by metagenomic data. Here we present the Metagenomic CRISPR Array Analysis Tool (MCAAT), a highly sensitive algorithm for finding CRISPR arrays in unassembled metagenomic data. It takes advantage of the properties of CRISPR arrays that form multicycles in de Bruijn graphs. We show that MCAAT reliably predicts CRISPR arrays in bacterial genome sequences and that its assembly-free graph-based strategy outperforms assembly-based workflows and other assembly-free methods on synthetic and real metagenomes. Our new approach will help to increase the diversity of known CRISPR-Cas systems and enable studies of spacer evolution within metagenomic data sets.

## Teaser

Schematic representation of a CRISPR locus (L: leader, DR: direct repeat, S_i_: spacers) and its De Bruijn graph representation forming a tangle with three distinct cycles (orange, purple and green) connected at the repeat nodes (blue).

## Introduction

CRISPR-Cas, short for Clustered Regularly Interspersed Short Palindromic Repeats and CRISPR-associated genes, systems constitute a bacterial adaptive immune system that protects their host against invading nucleic acids. Beyond this function in defense, some systems have been found to participate in cellular functions, e.g. to regulate gene expression (Mandin et al. 2007, Sampson et al. 2013). The diversity of CRISPR-Cas systems is large, and they are classified into two classes with several types and subtypes (Makarova et al. 2015, 2020). However, the diversity of known systems is limited because most of the studied systems come from cultured bacteria, which make up only a small fraction of all bacteria. Metagenomes could fill this gap because they are unbiased snapshots of a habitat at a certain time point. But the underlying data analysis comes with its own challenges and problems, with the assembly being the most critical. Usually, 10%–30% of the sequencing reads cannot be found in the assembled metagenomic contigs (Vollmers et al. 2017, Vosloo et al. 2021), which means a considerable loss of data.

CRISPR-Cas loci consist of the CRISPR array, the cluster of CRISPR-associated (Cas) genes, and the leader in front of the array. Although Cas genes differ significantly between the two classes, the CRISPR arrays always consist of direct repeats (DRs, 23–50 nt in length) interspersed with the so-called spacers. The latter typically also have lengths in the range of 23–50 nt. It is exactly this specific structure that led to their initial discovery (Ishino et al. 1987), and this structure is also the basis for their computational prediction.

There are many tools for the prediction and analysis of CRISPR-Cas systems [see the review in (Alkhnbashi et al. 2020)]. For CRISPR-Cas prediction, recently, CRISPRidentify was presented (Mitrofanov et al. 2021), which uses a strategy similar to CRISPRCasFinder (Couvin et al. 2018) that is based on repeat detection. The major difference is that CRISPRidentify uses a machine learning model to evaluate the quality of a candidate, which improves sensitivity and specificity compared to CRISPRCasFinder. Both tools are designed for genomic sequences, i.e. sequences of reasonable length. Now, let us turn to metagenomic data. For these, there are two fundamentally different approaches that can be used to predict CRISPR arrays. The first is to assemble the reads into contigs and then apply one of the aforementioned tools, where CRISPRCasFinder also provides a special flag (-meta) for this scenario. The second approach is to directly work on the reads, as, e.g. CRASS (Skennerton et al. 2013) is doing. As discussed in the review (Alkhnbashi et al. 2020), the performance of this highly depends on read length and quality because repeats are initially identified in single reads. The assembly-based approach suffers from the fact that the assembly graph is highly complex and needs to be simplified by methods such as tip removal and bubble merging (Li et al. 2015), resulting in a loss of data.

In order to get a comprehensive overview of CRISPR systems in metagenomic data, we have to make use of all the data, i.e. all sequencing reads, and to benefit from contiguous chains of overlapping reads in some sort of assembly. Metagenome assemblers typically make use of de Bruijn graphs (Compeau et al. 2011). A de Bruijn graph is constructed from *k*-mers of the sequencing reads, which form the nodes of the graph. Edges are formed between directly subsequent *k*-mers (nodes) of the same read. A fundamental property of a de Bruijn graph is that nodes are unique and thus read overlaps, which have equal *k*-mers, lead to longer paths in the graph, rather than new ones. Equal$\ k$-mers come not only from read overlaps but also from repeated sequence stretches, like DRs in CRISPR arrays. Interestingly, by construction, a repeat leads to a circle in the graph, and many repeats result in a multicycle. This fundamental feature is exactly what is the basis for our metagenomic CRISPR array analysis tool (MCAAT). As a proof-of-concept we show that it performs very well on genomic sequences and then present results on synthetic and real metagenomes, where we compare it to state-of-the-art tools.

## Materials and methods

### Graph construction

MCAAT uses the functions buildlib and read2sdbg provided by MEGAHIT (Li et al. 2015) to construct the graph. MEGAHIT is an ultrafast, memory-efficient assembler designed for next-generation sequencing (NGS) reads based on succinct de Bruijn graphs. For the construction of the graph, reads are split into subwords of length *k*, the so-called *k*-mers, which represent the nodes. Nodes with an overlap of length $k\ - \ 1$ are connected by directed edges.

By default, we use a *k*-mer size of 23, which corresponds to the minimum length of a repeat and spacer. The user can adjust this if needed. The construction includes counting of *k*-mer occurrences in all reads, and the resulting counts are used as the $Multiplicity( i )$ of the nodes. We use the term Multiplicity to emphasize the fact that it is a combination of the sequencing coverage and the frequency of repetition of a$\ k$-mer. MCAAT also makes use of the basic graph functions for traversal, label retrieval, and computation of incoming and outgoing edges provided by MEGAHIT. The number of incoming edges of node *i* is also called its $Indegree( i )$. Accordingly, the $Outdegree( i )$ is the number of outgoing edges of node *i*.

### Start node detection algorithm

The pseudocode in algorithm 1 shows how potential nodes for starting a cycle enumeration, called start nodes, are detected. Line 7 in algorithm 1 represents the filter criteria of two or more incoming edges and a multiplicity of at least 20.

**Figure:**
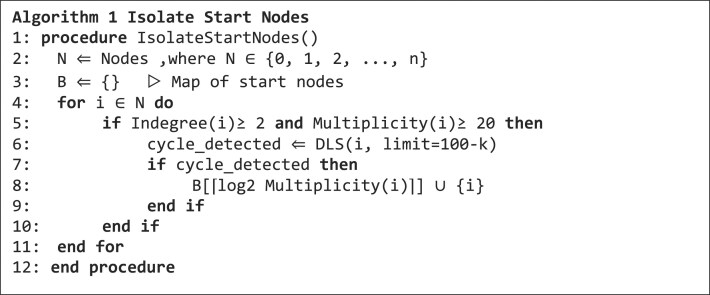


Any cycle within a larger graph needs to have at least one node with more than two incoming edges connecting it to the graph. For a multicycle, we furthermore expect this node to have a high $Multiplicity$, which can be interpreted as the product of repeat frequency and sequencing coverage. A threshold of 20, would be overcome by a two-repeat system with 10 times coverage or a 20-repeat system with single coverage as the extremes. Line 8 restricts the length of candidate cycles to a maximum length of $100\ - \ k$ nodes $( {DLS\ ( {i,\ \textit{limit} = 100 - k} )} )$. In line 9, the detected start nodes are grouped according to their rounded-up ${\mathrm{lo}}{{\mathrm{g}}}_2$ multiplicity, which is used for the distribution of parallel processes.

**Figure:**
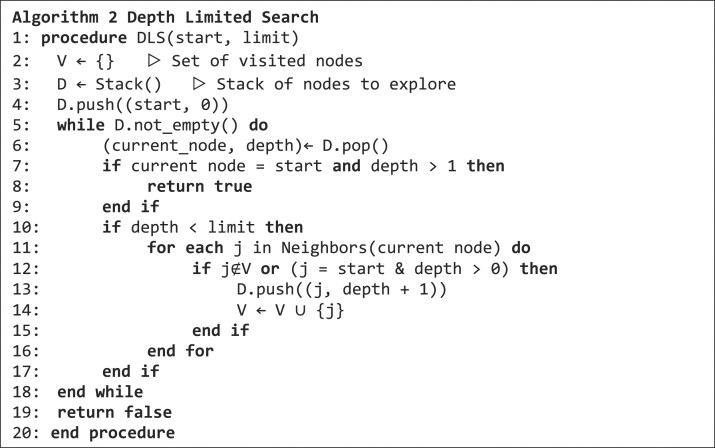


**Figure:**
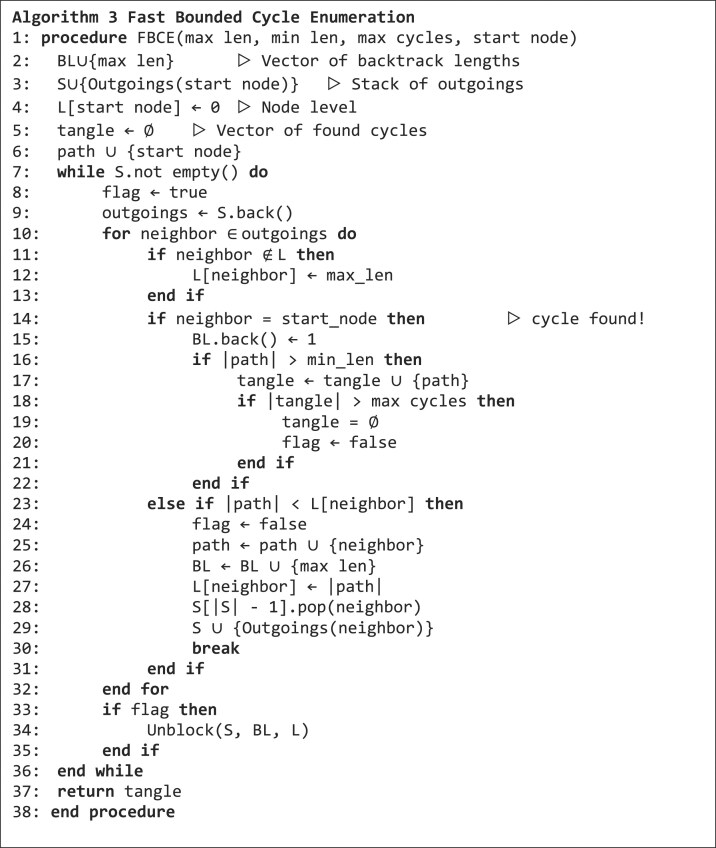


The pseudocode of the depth-limited search (DLS) algorithm is shown in algorithm 2. The DLS algorithm determines if there is a path from a given start node to itself with a specified length limit. If so, a cycle for this start node has been detected and the algorithm returns true.

### Fast bounded cycle enumeration

The core of our method is the fast bounded cycle enumeration algorithm (FBCE). It is an adapted version of the bounded-length cycles enumeration algorithm by Gupta and Suzumura (2021). We adapted the NetworkX (Hagberg et al. 2007) implementation of this algorithm to succinct de Bruijn graphs. Here, we can omit the construction of the so-called transpose graph because within succinct de Bruijn graphs incoming edges can be computed efficiently. Briefly, starting from a given node, the FBCE algorithm traverses the graph using depth-first search (DFS), with backtracking and additional bookkeeping (see pseudocode in algorithm 3). The procedure begins with the outgoing neighbors of the start node from the stack *S* and checks whether one of them is equal to the start node. If not, the neighbor is added to $path$ and its outgoing nodes to the stack *S*. These new nodes will then be investigated in upcoming iterations of the while loop. In case the neighbor is equal to the start node, a cycle is found and it is added to $tangle$. In case the number of cycles in a tangle exceeds the maximum cycle limit max cycles, the complete tangle is discarded. The data structures $BL$ and$\ L$ are needed to keep track of the level of nodes for backtracking and branching.

If a cycle is found or a dead end has been reached, the algorithm jumps back to the last unexplored branching point and starts (actually it continues) to search another cycle. For this, the data structures $S,\ BL$ and $L\ $need to be cleaned up, which is done with the unblock function in algorithm 4. This ensures that branches on any level are inspected for the possibility of forming a cycle.

**Figure:**
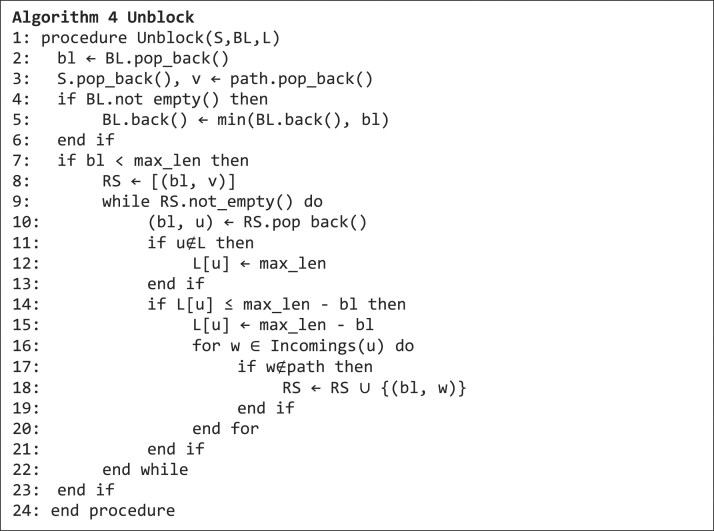


The executions of the FBCE algorithm on start nodes can be distributed across multiple threads to enable data-driven parallelism (Blanuša et al. 2023). Each thread gets a specific number of nodes for static processing.

### Benchmarking

We used 57 genomes from CRISPRCasDB and their annotated CRISPR arrays as the ground truth for benchmarking in the proof-of-concept and for the simulated metagenome. Furthermore, for the same 57 genomes from CRISPRCasDB, we used insilico-seq (Gourlé et al. 2019) to simulate 32 million sequencing reads based on the Illumina Hiseq error model using the command line call “iss generate –genomes ‘57genomes.fasta’ –coverage file ‘coverage.txt’ –model hiseq –output reads.” The accession numbers of the genomes, together with the coverage used for read simulation, can be found in the supplemental material. The resulting reads were assembled into contigs with megahit as follows: “megahit -1 ‘sim R1.fastq’ -2 ‘sim R2.fastq’ –prune-level 1 –bubble-level 1 -o ‘sim/assembly.’’ For the genome sequences in the proof-of-concept, MCAAT was used without the multiplicity filters for the start node and the cycle detection because *k*-mers from a single genomic sequence have a multiplicity of 1 except for repeated *k*-mers. CRISPRIdentify, CRASS, and CRISPRCasFinder-meta were used with settings resembling those of MCAAT as far as possible, resulting in the following command line calls for CRISPRIdentify “python3 CRISPRidentify.py –file ‘<input>.fasta’ –strand False –min len rep 23 –max len rep 50 –min len spacer 23 –max len spacer 50 –result folder ‘res’,” CRASS “crass ‘sim R1.fastq sim R2.fastq’ -o crass out,” and CRISPRCasFinder-meta “perl CRISPRCasFinder.pl -in <in>.fasta –meta -mr 23 -xr 50 -ms 23 -xs 50 -lMin 3 -out ‘res’.” For CRISPRidentify we considered bona-fide, possible and alternative candidates, and for CRISPRCasFinder-meta those with evidence levels 3 or 4.

For the real-world metagenome we used $100$ million reads from the marine metagenome dataset with the NCBI SRA run accession number SRR4028175. The results of the different tools were compared with a 90%similarity threshold (edit distance) to account for small variations between the predictions, e.g. due to slightly shifted repeat/spacer boundaries.

Computations were performed on Linux-based virtual machines with Ubuntu 20.04, 28 virtual CPUs and 256 GB RAM in an OpenStack cloud environment.

### Software availability

The source code of MCAAT as well as a docker container are available on GitHub (https://github.com/RNABioINfo/mcaat).

## Results and discussion

**Figure 1.**
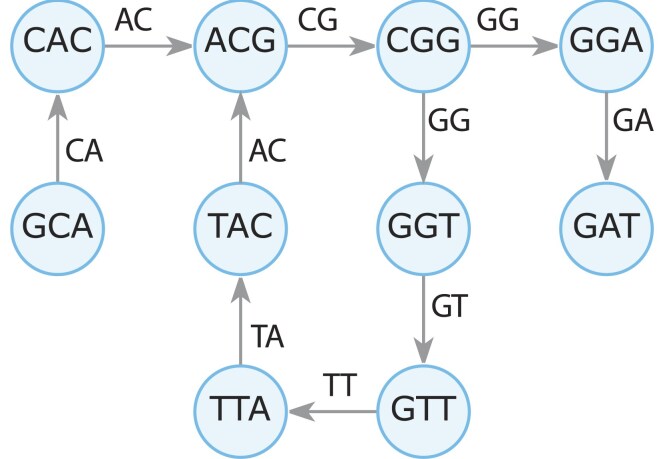
Example of a de Bruijn graph constructed from the read GCACGGTTACGGAT with k-mer length 3. Nodes are k-mers at every position of a DNA sequence and edges are overlaps between the nodes.

**Figure 2.**
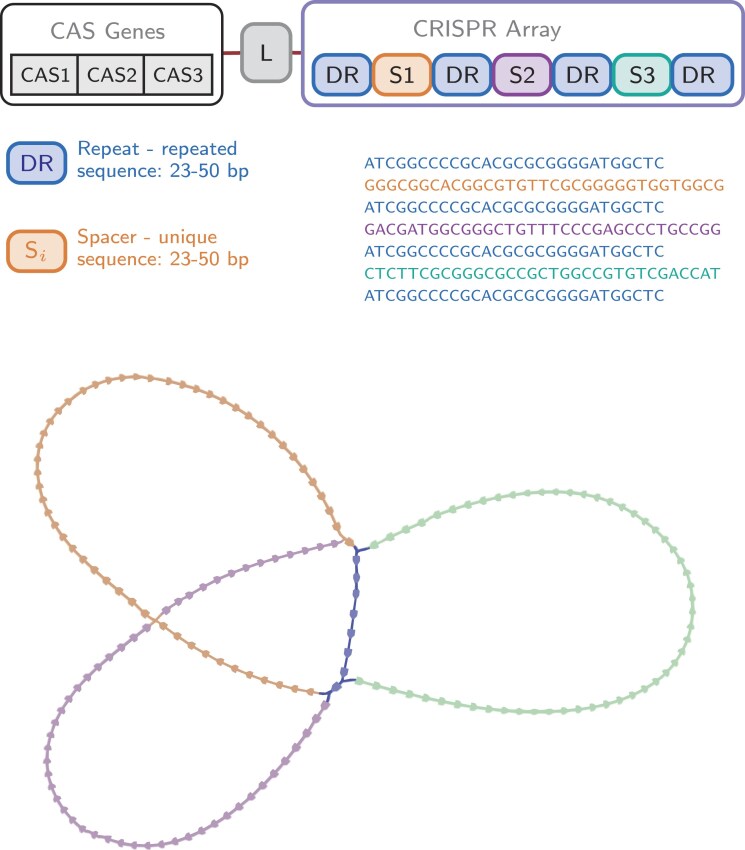
(Top) Schematic representation of a CRISPR locus. L: leader, DR: direct repeat, S_i_: spacers. (Bottom) De Bruijn graph built from the CRISPR array above forming a tangle with three distinct cycles (orange, purple, and green) connected at the repeat nodes (blue)

The basic data structure used by our algorithm is the de Bruijn graph. It is constructed from *k*-mers of the sequencing reads that form the nodes. The nodes are labeled with the *k*-mer sequence. The edges connect nodes that have an overlap of $k\ - \ 1\ $and are labeled by the union of the two *k*-mer sequences. An example is shown in Fig. 1. It nicely shows that the repeat “ACGG” (represented by nodes “ACG” and “CGG”) leads to a cycle in the graph. From this, we can easily anticipate that multiple repeats lead to multiple cycles. As a result, a CRISPR array (see Fig. 2) with *n* spacers and $n\ + \ 1$ repeats $DR + {S}_1 + DR + {S}_2 + DR + \ldots + {S}_n + DR$ will form a cluster of simple cycles (Johnson 1975) with$\ n$ cycles that have the nodes of the $DR$s in common. An example is shown in Fig. 2, and we refer to such a cluster as a tangle.

The example graph in Fig. 2 was built with a *k*-mer size of $21$. The repeats and spacers have lengths of 28 and 33 nt, respectively. It is important to note that by construction, the number of repeat nodes equals the repeat length minus the *k*-mer size. As a result, there are 28–21 =7 repeat nodes in the graph.

**Figure 3.**
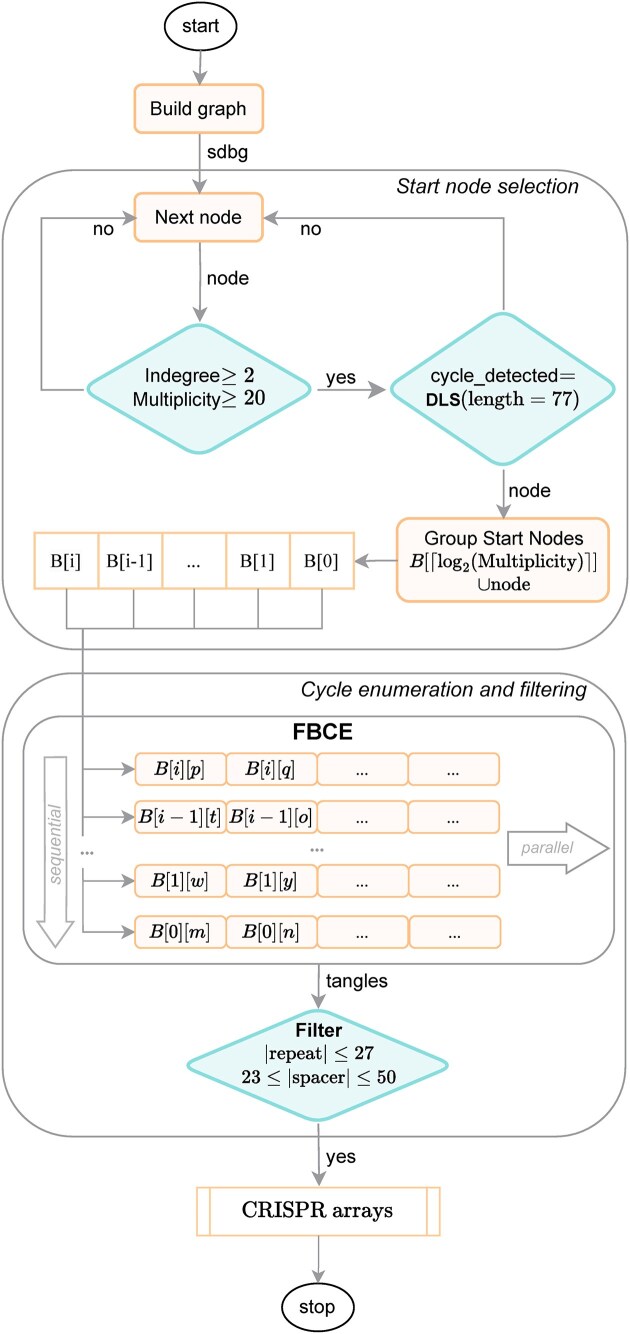
Overview of MCAAT. The algorithm starts by building the graph. Next, nodes are checked for their properties to serve as a start node and grouped based on similar multiplicity. Each group is then passed into the actual tangle detection in parallel processes. Finally, additional filters, e.g. for repeat and spacer length are applied and the resulting tangles transformed into repeat and spacer sequences that represent a potential CRISPR array.

Based on the above findings, the core idea of our algorithm is to identify and enumerate all cycles in a de Bruijn graph. An overview of the MCAAT workflow is given in Fig. 3.

The initial step is to construct the de Bruijn graph from the sequencing reads. Here we make use of the memory-efficient implementation, the so-called succinct de Bruijn graph (Muggli et al. 2017), provided by the metagenome assembly algorithm MEGAHIT (Li et al. 2015) and also use its graph construction routines. Essentially, finding a cycle simply means starting a path at a certain node and walking as long as you see the start node again, which means a cycle is found, or there is no further possible step, meaning no cycle found. In order for a CRISPR array to form a tangle in the graph, the *k*-mer size must be less than or equal to the repeat size, with 23 nt being the shortest known repeats. This rather small$\ k$-mer size, which is the default of our algorithm, results in huge graphs with billions of nodes that we have to deal with. This makes computational efficiency a central goal. In terms of memory, we achieve this by the use of the succinct de Bruijn graph representation, as mentioned before. For the computation, we introduce optimizations that take advantage of cycle properties, quantitative information, namely *k*-mer multiplicity, and the characteristics of CRISPR arrays. The different tweaks will be presented in the following paragraphs.

### Selecting start nodes

The number of start nodes determines the number of times the cycle-finding algorithm runs. Therefore, it is beneficial to reduce the number of start nodes. A cycle, as long as it is not an isolated cycle, will always have at least one special node. This one is connected to other parts of the graph (like the node “ACG” in Fig. 1) and therefore has two incoming edges. This is our first criterion for selecting start nodes.

The second criterion is related to the fact that in a CRISPR array, the repeat nodes are the ideal candidates to serve as start nodes. Repeat nodes have a rather high multiplicity, which is the number of times the nodes *k*-mer has been observed in a sequencing read. This multiplicity is a combination of sequencing coverage and repetitiveness and thus should be higher for repeat nodes. By default, we require a multiplicity of at least 20 for a start node, but this is a user-defined value.

The nodes that satisfy the two aforementioned criteria are then subjected to a quick cycle check. For this, we use a modified version of DFS called DLS (Kozen 1992). DLS requires a start and a target node to detect whether there is a path between those nodes at a given maximum length. We simply set the start and target nodes to be the same, and thereby detect cycles of a maximum length.

### Parallel computation

A straightforward way to improve the runtime of an algorithm is to make use of multiple CPU cores by parallel computation. For our algorithm, the problem is that it works on a single large data structure, the graph, and that all child processes need to access the same data structure at the same time. Due to the nature of the succinct de Bruijn graph, there is no efficient method to block certain parts of the graph or to split it into independent subgraphs. As a result, parallel processes can only be spread over the graph using a heuristic, which may result in two or more processes working on the same part and producing the same results. Nevertheless, we decided to implement such a heuristic because we think that the huge size of the graph and the large number of potential start nodes compared to the low number of parallel processes (typically between 16–64 cores per CPU) render this problem rather small. Furthermore, duplicate results can be easily removed in a post-processing step.

Tangles corresponding to CRISPR arrays can only be identified if the cycle enumeration starts from a repeat node. Unfortunately, the candidate start nodes are not guaranteed to be repeat nodes and thus can be, and are, actually frequently, spacer nodes. But we know that repeat nodes and spacer nodes from the same array differ substantially in their multiplicities, and if we manage to process the repeat nodes first, we can safely mark candidate start nodes that have already been found as part of a tangle to not be considered further. In order to separate repeat and spacer nodes from the same system, we group all start nodes by the rounded-up $lo{g}_2$ of their multiplicity. Please note that each group can still contain repeat and spacer nodes, but very likely only from different arrays. The groups are processed sequentially, starting with the highest multiplicity, and members within a group are processed in parallel and out of order (see Fig. 3). The latter decreases the likelihood of two processes working on the same part of the graph.

### Cycle enumeration and filtering

The central method of our algorithm is the enumeration of cycles. As mentioned above, repeats and spacers have lengths between 23 and 50 nt, and therefore, cycles corresponding to CRISPR arrays have lengths between 50 and 100 nt. Because a node itself has a length of *k* (default $k\ = \ 23$), cycles have lengths of 27–77 nodes. Our FBCE is based on the bounded–length cycle–finding algorithm by Gupta and Suzumura (2021), which we adapted to succinct de Bruijn graphs. Given an initial node, this algorithm is guaranteed to find all cycles up to a given length. For known CRISPR systems, the number of spacers usually ranges between 2 and 500 (Couvin et al. 2018). In case FBCE found only a single or more than 500 cycles for a single start node, these are not considered further.

**Figure 4.**
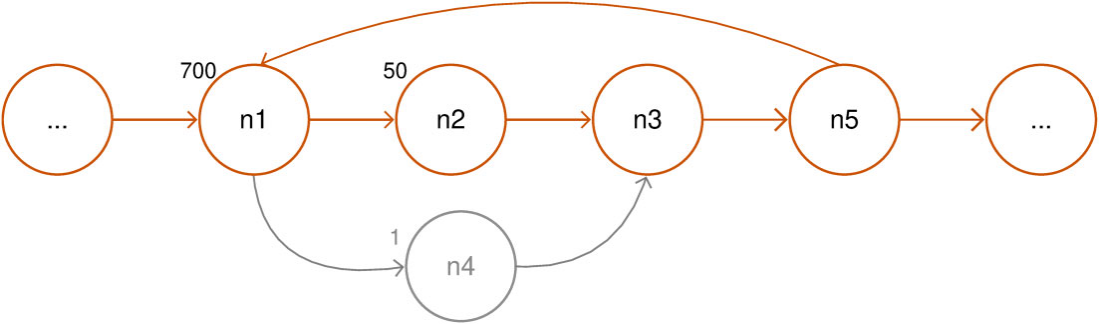
Example graph with two possible cycles: n1, n2, n3, n5, n1 and n1, n4, n3, n5, and n1. Numbers indicate the multiplicities of the nodes. Since the ratio of the multiplicity of n1 to that of n4 exceeds 500 the cycle n1, n4, n3, n5, and n1 would be excluded.

A special problem occurs due to sequencing errors that lead to additional branches (also called bubbles) and, within tangles, would also be detected as independent cycles as shown in Fig. 4. In order to prevent this, we only allow for paths where the multiplicity of the nodes is at least $\frac{{1\ }}{{500}}$th of the multiplicity of the start node. The reasoning behind this is that the start node is a repeat node and that its multiplicity should be at most 500 times larger than the multiplicity of a spacer node because we have at most 500 spacers/cycles. We are aware of the fact that we might lose actual systems that are low-abundance variants of other systems with this.

With the FBCE we are only able to limit the length of the full cycle but cannot control the length of the repeat and spacer parts independently. In order to do this, we need to map the nodes to be either part of the repeat or the spacer. We do this in a postprocessing step by analyzing the nodes of all cycles that belong to the same tangle. The nodes that are part of every cycle within a tangle are repeat nodes, and the others are spacer nodes. Please note that, depending on the position of the mutation, mutations in the repeats will result in either the detection of seemingly shortened repeats or the loss of individual repeats. We retain only those tangles where the number of repeat nodes (*r*) and the number of spacer nodes (*s*) satisfy the constraints: ($r\ \le \ 50\ - \ k$) and ($\mathit{ k}\ \le \ \mathit{ s}\ \le \ 50$).

To reconstruct the sequence of the CRISPR array, we first identify a repeat node that has no incoming edge from any other repeat node. This node represents the start of the repeat, and we reconstruct the sequence by following the outgoing edges to the other repeat nodes and through the spacer nodes back to the repeats and so on and so forth. Please note that for genome sequences, we can reconstruct the correct order of the spacers based on the node IDs because they are ordered according to their genomic position. For sequencing reads, it is currently not possible to reconstruct the correct order, but we are working on a solution for this.

### Proof of concept

Although MCAAT is designed for the analysis of raw metagenomic sequencing reads, it is suitable for the analysis of full-length genomes. With this in mind, we evaluated the performance in predicting known CRISPR arrays from genome sequences. We selected 57 genomes from CRISPRCasDB (Pourcel et al. 2019), with lengths ranging from 1 to 9 million base pairs and up to 101 CRISPR arrays per genome. For comparison, we also applied CRISPRidentify to the genomes and checked for correct predictions using the CRISPR arrays provided by CRISPRCasDB as the ground truth. Here, we used the repeat sequences as proxies for CRISPR arrays and, for better resolution, also compared the predicted spacers.

**Figure 5.**
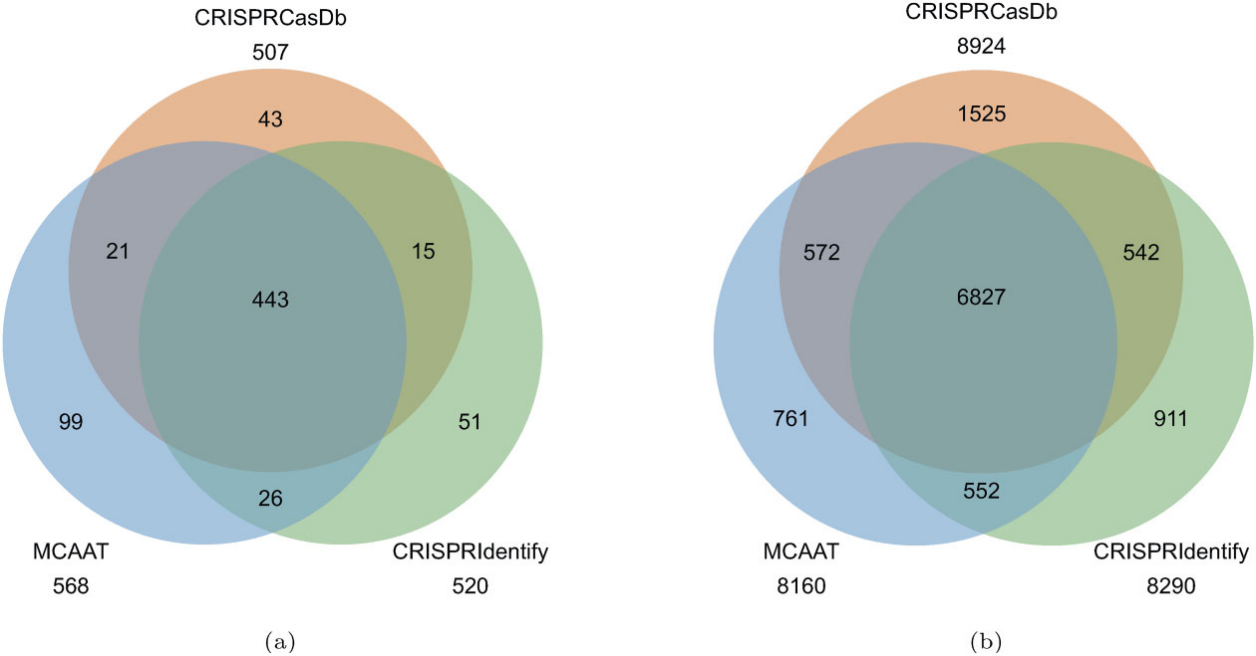
Venn diagrams of the (a) repeat-based (full arrays) and (b) spacer-based comparison of CRISPRidentify and MCAAT with CRISPRCasDB. Numbers below the tool names represent the total number of predicted repeats and spacers, respectively.

As shown in Fig. 5, MCAAT predicted 568 arrays and CRISPRidentify 520 from which 464 and 458 are true positives, respectively. Thus, MCAAT achieves a precision of 0.82 and a recall of 0.92, while CRISPRidentify has a precision of 0.88 and a recall of 0.90. It is important to note that we used CRISPRidentify with adapted MCAAT-like settings, so the numbers could be improved with some parameter optimization. Based on these results, we feel confident that MCAAT reliably predicts CRISPR arrays, although further optimization might also be possible here, but this is not the focus of our work.

### Simulated metagenome

To analyze the performance of MCAAT in a controlled setting on metagenomic data, we decided to use a simulated metagenome. This resembles at least to some degree the complexity of a real metagenome, while it still allows one to verify the predictions on a fixed ground truth. For this dataset, we simulated reads for 57 genomes from CRISPRCasDB with insilicoseq using different genome coverages. The genomes contain a total of 507 CRISPR arrays with 8924 spacers. It is important to note that many systems share the same repeat sequence such that there are only 312 unique repeat sequences. This has a significant impact on CRASS and MCAAT because both of them directly work with the raw reads such that it is impossible to distinguish identical repeats from different systems. The contig-based tools suffer from this to a minor degree because arrays with identical repeats could have been assembled into distinct contigs.

**Figure 6.**
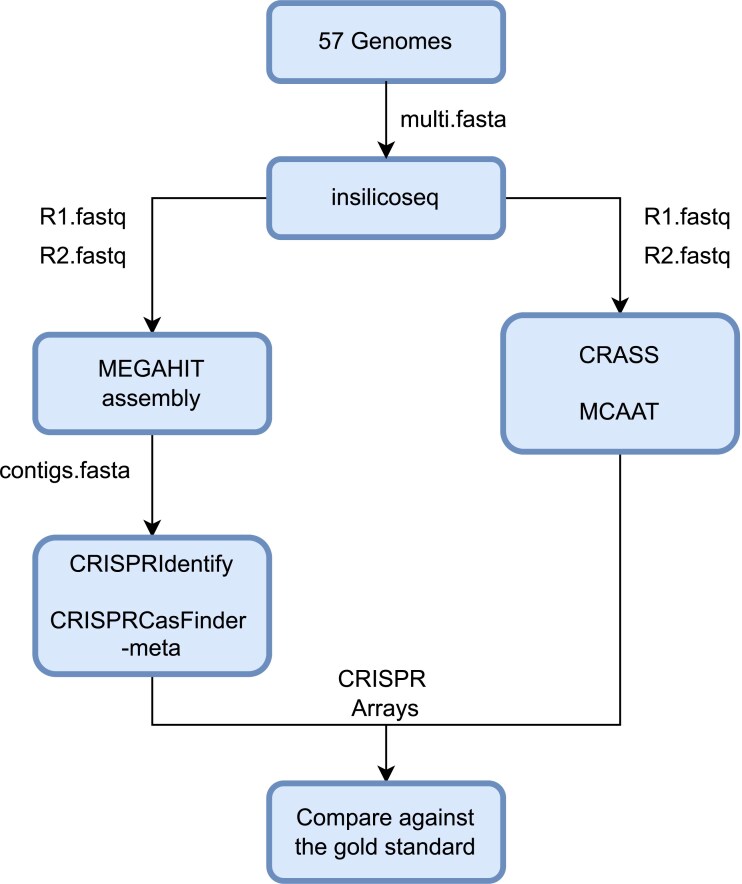
Overview of the benchmarking process for the simulated metagenome. 57 genomes from CRISPRCasDB were used as input for insilicoseq to generate paired-end sequencing reads based on an Illumina error model. CRISPRCasFinder-meta and CRISPRIdentify were used on contigs assembled with Megahit, while MCAAT and CRASS were used directly on the reads. The predictions were compared against CRISPRCasDB.

The simulated reads were directly analyzed with CRASS and MCAAT and assembled with Megahit into contigs that were analyzed with CRISPRidentify and CRISPRCasFinder-meta, which is a variant optimized for metagenomes, as shown in Fig. 6.

Table 1 summarizes the results of the benchmarking. MCAAT achieved the highest precision and recall for arrays and spacers. The slightly worse performance of MCAAT on the spacers is in our opinion mainly the result of arrays that have degenerate repeats. In the de Bruijn graph, these degenerate repeats are represented by distinct paths that are not part of the tangle and are thus not detected by MCAAT. As a result, the corresponding spacers are lost. Additional spacers may occur due to sequencing errors (reads were simulated with errors). A single nucleotide difference results in $k\ = \ 23$ different nodes that will form an additional cycle in the tangle and thus be reported as a distinct spacer. Simple checks for sequence similarity could be used to identify and remove these. This needs to be thoroughly investigated because also natural spacer variants could be removed by this. It is important to note that for the contig-based tools, CRISPRIdentify and CRISRPCasFinder-meta, the poorer performance is very likely due to shortcomings of the assembly. Furthermore, CRISPRIdentify is not designed for contigs but rather full genomes, which explains its slightly poorer performance compared to the metagenome adapted CRISPRCasFinder-meta.

**Table 1.** Prediction statistics for MCAAT, CRASS, CRISPRIdentify, and CRISPRCasFinder on the simulated metagenome.

	Arrays				Spacers				Runtime
Tool	Predicted	Matched	Precision	Recall	Predicted	Matched	Precision	Recall	[min]
MCAAT	374 (538)	291(455)	0.78(0.85)	0.93(0.9)	8602	6414	0.75	0.72	24
CRASS	295 (532)	141(198)	0.48(0.56)	0.45(0.39)	8310	3231	0.39	0.36	2
CRISPRIdentify	599	339	0.57	0.67	12 152	3872	0.32	0.43	640
CRISPRCasFinder -meta	525	375	0.71	0.74	9134	4043	0.44	0.45	137

For arrays, full repeat identity and for spacers full spacer identity was used as the matching criterion. In total, the reference genomes contain 507 arrays with 312 unique repeats and 8924 spacers. For MCAAT and CRASS, the numbers in brackets correspond to the number of arrays from the reference for which the repeat was found. This accounts for arrays with identical repeats, which are not distinguished by the two algorithms. Accordingly, for the recall we base the calculations on the number of unique repeats (312) and all arrays (507), respectively.

### Real-world metagenome

The previously used synthetic metagenome had the benefit of having a solid ground truth for benchmarking. However, its complexity is lower than that of a real metagenome. In order to get a realistic impression of the behavior and performance of MCAAT, we analyzed 100 Mio. reads of a publicly available metagenomic data set and compared it with CRISPRCasFinder-meta and CRASS. We omitted CRISPRIdentify because it is not designed for metagenomic data.

**Figure 7.**
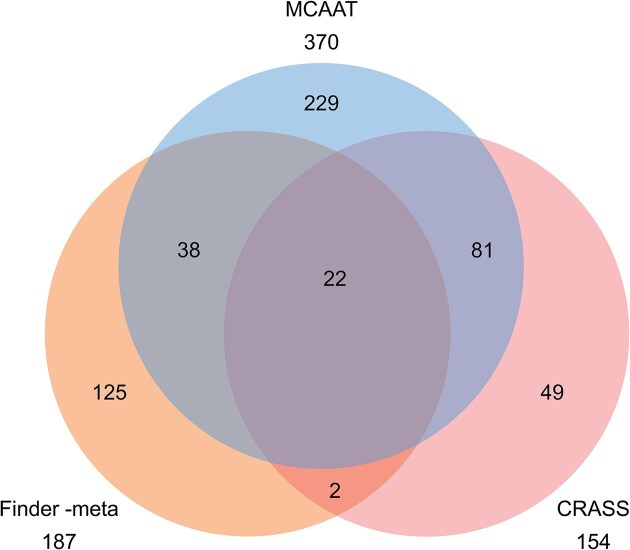
Venn diagram of CRISPR arrays predicted by MCAAT, CRISPRCasFinder-meta, and CRASS for the real-world metagenome.

The results are summarized in Fig. 7. MCAAT predicted the largest number of CRISPR arrays, and CRASS predicted the least. While the overlap between CRISPRCasFinder-meta and CRASS is small (24%, 13%, and 15%, respectively) MCAAT shares 32% and 67% of their predictions. The larger overlap between MCAAT and CRASS nicely reflects that both use raw reads for their analysis, while CRISPRCasFinder-meta uses assembled contigs. Why there is still a large number of predictions unique to CRISPRCasFinder is a question for future work. Based on the results for the synthetic metagenome, we expect that the predictions unique to both MCAAT and CRISPRCasFinder-meta carry a large fraction of true positives.

## Conclusion

With MCAAT, we present an efficient and reliable tool for the prediction of CRISPR arrays in metagenomic data. In its current implementation, it cannot distinguish identical repeats from different systems, but we already have an idea to resolve this via the use of co-occurrence information of spacer-derived *k*-mers in a sequencing read. Spacers of different systems cannot co-occur in a read, and thus, this information can be used to disentangle repeat-identical systems. In a similar manner, this information can also be used to derive the relative order of spacers, which is, e.g. of interest in evolutionary and host-virus coevolution studies [reviewed in (Westra et al. 2016)]. In this context, MCAAT could serve as a valuable source for already existing CRISPR array analysis and visualization tools, such as CRISPRtrack (Lam and Ye 2019), CCTK (Collins and Whitaker 2023), and SpacerPlacer (Fehrenbach et al. 2024).

The graph-based implementation offers the potential for further extensions, e.g. to look for Cas genes in the neighborhood of an identified CRISPR array. This can be achieved by depth-first walks going backwards from a repeat node until a Cas-gene-specific sequence signature is found. Similarly, CRSIPR leader sequences could be identified, e.g. with the HMM-based approach of CRISPRleader (Alkhnbashi et al. 2016). Furthermore, we can also identify potential protospacers, which would be paths in the graph that share nodes with the spacer part of a cycle. These paths can be assembled into contigs and checked for their origin, i.e. if they are viral sequences or something else. This would help to analyze the question of spacer origin to greater depth than it is currently possible based on sequenced bacterial and viral genomes (Shmakov et al. 2020). Furthermore, protospacer contigs can also be used to identify possible protospacer adjacent motifs (PAMs).

These extensions, together with further optimizations, will enable large-scale studies on the number and diversity of CRISPR-Cas systems in natural habitats. Furthermore, it will enable evolutionary studies on shorter and longer time scales, focusing on the ongoing battle of bacteria and archaea against viruses and other invading nucleic acids. Perhaps even more interesting could be the cases, where the identified systems harbor spacers with unusual targets, shedding light on so far unknown defense or regulatory functions.

## Supplementary Material

uqaf016_Supplemental_File
